# We Need to Talk About Racism—In All of Its Forms—To Understand COVID-19 Disparities

**DOI:** 10.1089/heq.2020.0069

**Published:** 2020-09-25

**Authors:** Adrienne Milner, Berkeley Franz, Jomills Henry Braddock

**Affiliations:** ^1^ Department of Health Sciences, Brunel University London, Uxbridge, United Kingdom.; ^2^ Department of Social Medicine, Heritage College of Osteopathic Medicine, Ohio University, Athens, Ohio, USA.; ^3^ Department of Sociology, University of Miami, Coral Gables, Florida, USA.

**Keywords:** racism, COVID-19, health disparities, discrimination

## Abstract

**Purpose:** Racism is an essential factor to understand racial health disparities in infection and mortality due to COVID-19 and must be thoroughly integrated into any successful public health response. But highlighting the effect of racism generally does not go far enough toward understanding racial/ethnic health disparities or advocating for change; we must interrogate the various forms of racism in the United States, including behaviors and practices that are not recognized by many as racism.

**Methods:** In this article, we explore the prevalence and demographic distribution of various forms of racism in the United States and how these diverse racial ideologies are potentially associated with racialized responses to the COVID-19 crisis.

**Results:** We find that among white Americans, more than a quarter express traditional racist attitudes, whereas more than half endorse more contemporary and implicit forms of racist ideology. Each of these types of racism helps us explain profound disparities related to COVID-19.

**Conclusions:** Despite a robust literature documenting persistent patterns of racial disparities in the United States, a focus on the role that various forms of racism play in perpetuating these disparities is absent. These distinctions are essential to realizing health equity and countering disparities in COVID-19 and other health outcomes among people of color in the United States.

## Introduction

Many public health scholars, activists, and leaders were not surprised to learn of dramatic racial and ethnic disparities in COVID-19 morbidity and mortality.^1^ Racial and ethnic minorities experience many chronic and acute illnesses at vastly higher rates than their white counterparts in the United States and die years earlier due to longstanding inequality in the resources necessary to protect health. COVID-19 merely follows the existing pattern of racial/ethnic health disparities that sets the United States apart from many other countries. Public health scholars have made vital contributions to understanding these disparities, in particular documenting how segregation, institutional racism, and racial bias have contributed to excess morbidity and mortality for Americans of color. However, racism takes a myriad of forms, and these nuances have particular implications for how we address COVID-19 and other health disparities.^2^

Racism can be defined as the ways in which individuals are privileged or disadvantaged, overtly and covertly, on a macro and micro level, based on perceived biological and cultural differences between groups, most often relative to skin color. Based on this definition, racism can result from the actions of individuals or institutions and can unfold in ways that are both intentional and unintentional. These distinctions may seem unnecessarily precise, but they have enormous implications for the promotion of health equity. Although many Americans still envision racism as individual prejudice, reminiscent of the Jim Crow era, racism has evolved to take on many different forms, some of which may be barely recognizable as racism. Each form has critical implications for understanding and addressing public health challenges such as COVID-19.

Sociologists have developed various theories of racism that seek to explain how and why whites have continued to preserve their power.^3–10^ These studies have helped to explain the shift from old-fashioned Jim Crow era biological explanations of racial inferiority to modern justifications for blacks' subservient group position. In addition, they have contributed to developing a better understanding of the harms of contemporary racial ideology that downplays the existence of racism at all.

Building on these theories, Adrienne Milner developed an operational typology of white racial ideology that encompasses *old-fashioned racism*—characterized by the belief that blacks are inherently inferior to whites; *institutional/systemic racism—*inequities rooted in the practice of social and political institutions*; symbolic racism*—the belief that black people are responsible for their disadvantaged social position; *laissez-faire racism*—the notion that blacks' cultural inferiority is to blame for racial inequality; and *color-blind racism*—the belief that racism and discrimination are no longer a problem and that equal opportunity exists.^11^ Understanding how different types of racial ideology may impede efforts to achieve equity is a necessary step to counteracting the effects of racism and developing antiracist ideology that reduces health disparities such as those found with COVID-19. In this article we explore (1) the prevalence and demographic distribution of each ideological type and (2) how these diverse racial ideologies are associated with COVID-19 disparities.

## Methods

To examine the prevalence and demographic distribution of each ideological type, we analyzed data on white participants from the American National Election Studies (ANES) 2016 Time Series Study (*N*=2530). This survey of United States-based citizens aged 18 years and older (ANES 2016 was carried out between September 7 and November 7, 2016, and from as many as possible of the same participants between November 9, 2016, and January 8, 2017. Though old-fashioned, institutional, symbolic, laissez-faire, and color-blind theories of racism are discussed and analyzed separately in this article, this does not imply that these are mutually exclusive categories; rather, individuals can adopt any number of orientations on varying issues, and at different times over their life course.

A combination of factor analysis and scaling techniques was employed to develop constructs based on the theoretical rationale for each type of racism: the *old-fashioned racism* scale consisted of three indicators of how warm or cold respondents feel toward black, Hispanic, and Asian people (Cronbach *α*=0.89). The *institutional racism* scale consisted of four indicators of how much influence respondents perceive white, black, Hispanic, and Asian people to have in politics (Cronbach *α*=0.82). The *symbolic racism* scale consisted of four of the eight indicators used in Henry and Sears's Symbolic Racism 2000 Scale (Cronbach *α*=0.86).^12^ The *laissez-faire racism* scale consisted of six indicators about whether respondents feel that blacks, Hispanics, and Asians were lazy or violent (Cronbach *α*=0.79). The *color-blind racism* scale consisted of three indications of whether respondents think there is discrimination against blacks, Hispanics, and Asians (Cronbach *α*=0.79). Because this analysis only employed secondary data, this study was not considered human subjects research by our institutional research boards.

## Results

The majority of white Americans possess more racist than less racist attitudes (Table 1). Although only roughly 25% of the sample expressed more old-fashioned racist attitudes, for all other types of racism, more than half of the respondents had scores representing racist attitudes. We found that white Americans were most likely to hold racial attitudes aligned with contemporary theories of racism such as symbolic racism (67.9% of the sample), followed by color-blind racism (64% of the sample), and laissez-faire racism (63.2% of the sample).

**Table 1.** Demographic Profile of Different Racial Ideologies Among White Americans (*N*=2530): Means and Percentages for Control Variables of Respondents with More Racist than Antiracist Attitudes

Percent of sample	Old fashioned 25.3	Institutional 52.5	Symbolic 67.9	Laissez-faire 63.2	Color-blind 64.0
Age (mean)	47.2	50.8	50.4	48.2	50.1
Education (% college degree)	24.0	28.3	27.3	32.7	33.3
Evangelical protestant (%)	17.3	21.0	20.5	17.8	18.8
Household income (mean)	$56,925	$59,225	$59,225	$59,992	$62,292
Male (%)	50.1	50.4	48.8	48.5	50.1
Married (%)	54.8	59.8	59.4	58.6	61.4
Democrat (%)	22.3	16.4	17.8	25.1	19.9
Independent (%)	34.4	34.3	35.1	35.5	34.2
Republican (%)	38.9	46.0	43.6	35.5	42.7

This profile highlights that those holding old-fashioned racist attitudes on average have a lower socioeconomic status in terms of education and household income than those exhibiting other types of racist attitudes. Conversely, those adopting color-blind racist attitudes tend to have a higher socioeconomic status than those who exhibit other types of racist attitudes. Across all types of racism, self-identified Republicans are much more likely to hold more racist attitudes than Democrats, with independents falling in the middle. Republicans are nearly three times as likely to adopt institutional racist attitudes, and more than two times likely to adopt symbolic and color-blind racist attitudes than their Democrat counterparts.

## Discussion

How do different forms of racism shape COVID-19 disparities?

### Old fashioned

It is logical that old-fashioned racism was the least adopted form of racism by white participants in our analysis. Before World War II, old-fashioned, or Jim Crow, racism was the dominant racial ideology in America. Characterized by the belief that blacks are inherently inferior to whites, old-fashioned racism promotes overt endorsement of nonegalitarian beliefs, such as support for racial segregation and open discrimination.^13^ Old-fashioned racism has become less prominent, although this form of racist ideology is still expressed by more than a quarter of the white population.

Although old-fashioned racism is no longer the dominant racial ideology in the United States, in the presence of a health threat brought forth by COVID-19, this type of racism seems to have been activated. Reports of harassment toward people of East Asian descent have skyrocketed. In addition to harassment and violence, Asian Americans are currently facing a disproportionate rate of unemployment in New York that stems at least partly from customers avoiding Asian restaurants and businesses.^14^ Old-fashioned racist attitudes have also been evident in terms of responses to COVID-19 infection rates among black Americans with many white Americans blaming genetic differences, rather than social conditions for differences in outcomes.^15^ In addition to the short-term effects of discrimination, activation of the stress process^16^ and unemployment and poverty^17^ are strongly linked to a host of chronic disease outcomes, suggesting that COVID-19 might not only reveal existing disparities, but also create new patterns of racial inequality altogether.

### Institutional and systemic

Not all racism occurs on the individual level, as racist ideology has shaped the structure and operation of major social institutions in ways that limit opportunity and perpetuate racial inequality. Racial ideas and ideology shaped US law, and through historical and governmental processes, functioned to create and dominate various racial groups. For example, scholars point to real estate practices such as redlining, voting laws, and educational policies that limit economic, political, and social opportunity for nonwhite Americans coming into contact with these institutions. This form of racism is particularly insidious because it is difficult, if not impossible to pinpoint the individual prejudice or racial ideology that is responsible for discrimination. As a result, this type of racism is often invisible to those not affected by it, and through downplaying its existence, whites can perpetuate existing inequality.

One of the early hypotheses of why COVID-19 has been associated with higher morbidity and mortality in predominantly black communities is that black Americans are more likely to have chronic diseases that are risk factors for severe COVID-19 infection.^18^ Disparities in chronic disease have been tied consistently and causally to historical processes such as real estate practices, mass incarceration, and discrimination in educational settings.^19,20^ Redlining, for example, created widespread disinvestment in communities of color, and contributed to growing neighborhood racial segregation.^21^ Today you can view old redlining maps, which guided where banks were willing to invest, in comparison with rates of asthma, diabetes, and hypertension and see the historical connection between decades of discrimination and chronic illnesses that are primary risk factors for COVID-19 severity.^22^ Examples of how institutional discrimination shapes the current distribution of chronic health disparities are too numerous to count, but result from the proximity of nonwhite neighborhoods to factories and other environmental pollutants,^23^ poor housing stock that predisposes children specifically to lead and respiratory contaminants,^24^ and exposure to discrimination within white institutions that is associated with chronic stress and disease.^25,26^ The composition of America's communities, especially in urban areas, remains largely segregated and these patterns also shape where COVID-19 testing locations are present and the accumulation of political power that would allow for shared decision making in responses to the pandemic.^27^

### Symbolic

Symbolic racism involves the belief that black people are themselves responsible for any disadvantages faced. This contemporary version of racism includes downplaying the existence of historic and systemic racism and instead focuses on black people's perceived lack of ability to succeed compared with other racial minorities. This belief system can help us understand why many Americans are resistant to policies that would ameliorate racial inequality, especially in terms of widespread health disparities.^28^ For example, disparities exist in insurance rates by race in the United States, but policies such as the Affordable Care Act (ACA) were heavily criticized for providing social welfare benefits without addressing the individual behaviors that were at the heart of insurance disparities.^29^ Lack of insurance is associated with poor health care access and the progression of chronic diseases that are primary risk factors for severe COVID-19 infection. Americans without health care coverage, who are disproportionately nonwhite, are not only at higher risk for infection from COVID-19, but also face considerable economic barriers if they need medical treatment in a hospital. The lack of support for policies to redress centuries of disparities in health care access is a direct result of racial stereotypes adopted within the framework of symbolic racism such as believing that black Americans themselves, rather than historic and persistent racism, are responsible for disadvantage.

Beyond the health effects of COVID-19, Americans face a yet unknown economic future as businesses and local governments begin to recover from the effects of a near total economic shutdown. Unemployment due to COVID-19 is concentrated among nonwhite Americans, especially in the cities hardest hit by the virus. Again, COVID-19 did not create racial disparities in either health or wealth, but existing inequality makes it harder for people of color to weather the effects of COVID-19. Whites hold ∼10 times the wealth of black Americans and these resources are critical to surviving an economic recession.^30^ Although the wealth gap is certainly a product of old-fashioned racism and discrimination built into systems and institutions, it is sustained by symbolic racism and the unwillingness of white Americans to support policies that target racial inequality.

### Laissez-faire

Laissez-faire racism is the notion that blacks' cultural inferiority is to blame for the black–white gap in socioeconomic standing. Bobo and Smith argue that laissez-faire racism differs from symbolic racism in that it is rooted in a historical analysis of the changing economic politics of race.^31^ Laissez-faire racists do not feel that black people and other racial minorities are as capable as white people, especially in terms of employment because of the racial stereotypes they hold. Specifically, they feel that black people and other racial minorities should not be in positions that are thought to require intelligence, hard work, and composure or that provide racial minorities with power and responsibility over whites.

Laissez-faire racism is exemplified in people of color disproportionately working in jobs that are deemed essential or that require significant risk of exposure to COVID-19.^32^ Jobs with less autonomy also activate the stress process in ways that make persons more vulnerable to severe illness and limit the ability to stay home during a pandemic or receive sick leave if one does become ill.^33^ In addition, laissez-faire racism results in a lack of racial minorities in decision-making positions. That is to say that white people, both as leaders in corporate and political settings, have been tasked with deciding when it is “safe” for their employees and constituents to end lockdown protocol. The result is that racial minorities who may also be at the most risk for COVID-19 severity are forced to go to work, use public transportation, and even vote in person against their personal safety preferences.^34^

### Color-blind racism and racial apathy

Color-blind racism refers to a new racial ideology rooted in apathy and disagreement with government policy, ensuring the fair treatment of racial minorities. Bonilla-Silva^9^ argues that through the emergence of color-blind racism, whites have developed powerful explanations for contemporary racial inequality that exculpate them from any responsibility for the status of people of color. Color-blind racism allows whites to maintain their advantages without having to articulate at whose expense. For example, whites who say, “I don‘t believe in giving black people access to health care—they should get it themselves but are too lazy to get a job” is thought to reflect a laissez-faire racist ideology, an older form of cultural racism. In contrast, whites who say, “I do not believe in giving black people access to health care because no one should get anything because of their race” could reflect a new form of modern or color-blind racism (adapted from affirmative action example given in Bonilla-Silva^9^).

Although the emphasis on cultural stereotypes epitomizes symbolic racism, a more covert, and perhaps insidious, form of racism operates among Americans who simply do not think that race any longer affects social, health, or economic outcomes. The result is racial apathy, or the feeling that race no longer is of consequence and as such the topic of racism can be avoided.^10^ Epidemiologists have uncovered patterns of premature aging or “weathering” among nonwhite Americans who experience subtle forms of interpersonal aggression by people who claim to not be acting with prejudice. This very type of chronic stress, however, has been linked to a vast array of illnesses, from premature labor to hypertension, and heart disease—all of which may exacerbate the severity of COVID-19 infection and mortality from the disease.

Evidence suggests that individuals who do not view themselves as racist often act in discriminatory ways, sometimes to their own surprise. There is growing evidence that physicians may unintentionally favor white patients and provide disproportionate time, warmth, and medical resources.^35^ Past studies have shown that black patients routinely wait longer for medical care, receive fewer pain medications, and are referred less often to the transplant list.^36–38^ In the context of COVID-19, unconscious or implicit bias likely shapes decisions for who gets medical treatments, especially when resources are scarce, such as being admitted to a hospital or receiving a ventilator. Other examples may extend to the receipt of personal protective equipment (PPE) for nonwhite medical professionals and essential workers or on who receives COVID-19 tests if presenting with symptoms. A teacher from New York is just one example of a black patient meeting the criteria for a test yet not receiving one until her illness had progressed to a point that it eventually killed her.^39^

## Health Equity Implications

Determining the manner in which different types of racism shape health disparities is essential for counteracting the effects of modern racist ideology and promoting health equity. Although racism is certainly at the heart of inequality in outcomes related to COVID-19, many white Americans do not see themselves as participating in or perpetuating a racist social system. As such, it is necessary to elaborate the myriad ways that racism persists in the United States and contributes to social and health inequality. It is simply not enough to state that racism exists.

True antiracism and the elimination of health disparities will require interrogation of the different forms racism takes. Old-fashioned, systemic, symbolic, laissez-faire, and color-blind racist ideology, we argue, each help us understand how and why whites have continued to maintain their privilege and health advantage—especially during a global pandemic. Our descriptive analysis (1) illustrates that contemporary forms of racism are especially prevalent among white Americans and (2) provides examples of how these diverse racial ideologies are potentially with racial/ethnic disparities related to COVID-19. Thus, there is an apparent need to go beyond studying racial health disparities using broad classifications of racism and also focus on how specific types of racism contribute to differential outcomes. Only then can we develop antiracist countermeasures that address the complex ways in which various types of racism challenge the promotion of health equity in the United States.

## Abbreviation Used

ANES: American National Election Studies
